# Comparative impact of direct oral anticoagulants and vitamin K antagonists on cognitive function in atrial fibrillation patients: a systematic review and meta-analysis

**DOI:** 10.25122/jml-2025-0071

**Published:** 2025-09

**Authors:** Ali Eisa Alkathiri, Hind Abdulrahim Alsulami, Lamis Atef Alshehri, Rowaid Khalid Aljabri, Shahad Ahmad Alhikan, Rahaf Mohammed Alanasari, Reem Matoqq Almalki, Shahad Eesa Alsulami, Sara Naif Alfaheid, Yasmeen Fahd Alabbas, Mohammed Bakri Alnashri, Fatimah Mohammed Alaqwal, Ilaf Mahmud Siraj, Lama Jamel Alosaimi, Amr Ahmed Fouad

**Affiliations:** 1 Faculty of Medicine, Al-Baha University, Al-Baha, Saudi Arabia; 2 Faculty of Medicine, King Abdulaziz University, Jeddah, Saudi Arabia; 3 Faculty of Medicine, Tabuk University, Tabuk, Saudi Arabia; 4 College of Medicine, Umm Al-Qura University, Mecca, Saudi Arabia; 5 College of Medicine, King Faisal University, Al-Ahsa, Saudi Arabia; 6 Faculty of Medicine, Jeddah University, Jeddah, Saudi Arabia; 7 College of Medicine, Imam Abdulrahman Bin Faisal University, Dammam, Saudi Arabia; 8 College of Medicine, Vision Colleges, Riyadh, Saudi Arabia; 9 College of Medicine, Ibn Sina National College, Jeddah, Saudi Arabia; 10 College of Applied Medical Science, Taif University, Taif, Saudi Arabia; 11 Department of Pharmacology and Therapeutics, Faculty of Medicine, Al-Baha University, Al-Baha, Saudi Arabia

**Keywords:** atrial fibrillation, direct oral anticoagulants, vitamin K antagonists, warfarin, cognitive function

## Abstract

The aim of this systematic review and meta-analysis was to compare the impact of direct oral anticoagulants (DOACs) and vitamin K antagonists (VKAs) on cognitive function in patients with atrial fibrillation. In August 2024, multiple electronic databases were searched following a two-phase screening strategy. Meta-analyses were performed using RevMan version 5.4. Effect estimations were presented as risk ratios (RRs) with 95% confidence intervals (CIs) for dichotomous outcomes. Eleven studies published between 2018 and 2024 were included in this research. Cognitive function assessments show minimal changes between the groups. At 5 years, DOACs were associated with lower rates of intracerebral bleeding (0.9% vs 1.4%) and mortality (17.6% vs 25.1%), while the incidence of all-cause dementia was identical in both groups (3.9%). At 10 years, DOACs continued to demonstrate benefits, with a reduced incidence of vascular dementia (0.7% vs 1.2%), lower all-cause mortality (23.2% vs 34.2%), and a decreased incidence of all-cause dementia (3.3% vs 4.7%). Meta-analysis results indicate a significantly lower risk of stroke or systemic embolism with DOACs (pooled RR = 0.30; 95% CI, 0.26–0.34), while the pooled risk for all-cause death showed no significant difference between the groups (pooled RR = 0.44; 95% CI, 0.16-1.16). DOAC users exhibited a lower incidence of all-cause dementia and fewer adverse events, particularly in long-term follow-ups. As a result, DOACs may provide a safer profile and a minor cognitive advantage over VKAs.

## INTRODUCTION

One of the most common types of arrhythmias is atrial fibrillation (AF). Currently, the treatment for patients with AF mainly incorporates oral anticoagulants (OACs). OACs have been linked to cognitive decline in patients with atrial fibrillation, according to studies; however, relevant evidence is lacking [1]. Direct oral anticoagulants, commonly referred to as blood thinners, are medications that prevent or reduce blood clotting, thereby prolonging the clotting time. Some occur naturally in blood-feeding animals, such as leeches and mosquitoes, which help keep the bite area unclothed long enough for the animal to get some blood [2]. As a class of drugs, anticoagulants are used to treat disorders that cause excessive blood clotting. Many take oral anticoagulants in tablet or capsule form, and different intravenous anticoagulants are used in hospitals. Some anticoagulants are used in medical equipment, such as specimen tubes, blood transfusion bags, cardiopulmonary bypass machines, and dialysis equipment. One of the first anticoagulants, warfarin, was initially approved as a rodenticide. Anticoagulants are closely related to antiplatelets and thrombolytics in their influence on various blood clotting pathways. Superficially, antiplatelet drugs inhibit platelet aggregation, while anticoagulants inhibit specific pathways in the coagulation cascade that may occur after initial platelet aggregation, ultimately leading to the formation of fibrin and stable platelet aggregation products [3].

AF is characterized by the sudden occurrence of abnormal electrical impulses in the atria that override the regulation of the heart’s natural pacemaker. In AF, normal and coordinated atrial electrical activity is lost, resulting in irregular fibrillatory waves and rapid, disorganized atrial contractions [4]. This atrial electrical disorder is a serious condition that causes atrial myocardial cells to contract rapidly and irregularly, producing symptoms such as palpitations, syncope, shortness of breath, and fatigue [1]. The risk of AF increases with age, particularly in individuals over 70 years, and in those with lifestyle-related conditions such as hypertension, diabetes, and obesity. With the global aging population, the prevalence of AF is expected to rise, with projections estimating more than a 60% increase in incidence by 2050. AF significantly impairs patients’ quality of life and is associated with serious complications, including stroke, heart failure, cognitive decline, and dementia, all of which contribute to increased morbidity and mortality [5].

A systematic review found that AF raises the risk of Alzheimer's disease (AD), all-cause dementia, vascular dementia, and cognitive impairment (RR = 2.2; 95 % CI = 1.4-3.5; *P* = .05). Management of AF primarily includes rate control, restoration of sinus rhythm, and anticoagulant therapy, with long-term anticoagulation required for patients with permanent AF [6].

Most patients with AF are treated primarily with anticoagulant medications, particularly oral anticoagulants. Vitamin K antagonists (VKAs) and direct oral anticoagulants (e.g., dabigatran, rivaroxaban, and apixaban) are generally utilized in the treatment of AF with excellent results [5].

Aspirin and clopidogrel are antiplatelet agents that are widely used in clinical practice and were therefore included in this review for comparison. Anticoagulation has been suggested to reduce the risk of dementia, whereas abnormal hemostasis may contribute to dementia development [6]. Several systematic reviews have reported that OACs significantly decrease the risk of cognitive decline. For example, Lee *et al*. found that non–vitamin K oral anticoagulants (NOACs) were more effective than warfarin in reducing dementia risk (OR = 0.65; 95% CI, 0.34–1.25; *P* < .05). Similarly, another study reported that OAC use was associated with a reduced risk of dementia (RR = 0.79; 95% CI, 0.67–0.93; *P* < .05) [7]. Cheng *et al*. further demonstrated that, compared with warfarin, NOACs lowered the risk of cognitive impairment (HR = 0.51; 95% CI, 0.37–0.71; *P* < .05). Nonetheless, the relative effectiveness of different anticoagulants in reducing cognitive decline remains uncertain [7].

Guidelines strongly recommend oral anticoagulation as the foundation for stroke prevention in AF. On the other hand, there is insufficient evidence to suggest that taking anticoagulants increases the risk of cognitive impairment. A new report showed that warfarin-treated AF patients were at a high risk of dementia due to under-or over-anticoagulation. An observational study also indicated that delays in initiating warfarin therapy could increase dementia risk in AF patients without a prior history of cognitive decline. Hence, it is conceivable to speculate that warfarin could add to the protected mental capability because of the stroke counteraction. Non-vitamin K antagonist oral anticoagulants, which selectively inhibit either thrombin or factor Xa, have been proposed as an optimal alternative to warfarin, given their effectiveness in thromboembolism prophylaxis and their lower risk of bleeding in AF patients [7].

## MATERIAL AND METHODS

### Literature search

This systematic review and meta-analysis were conducted in accordance with the Preferred Reporting Items for Systematic Reviews and Meta-Analyses (PRISMA) guidelines. The review protocol was registered in the PROSPERO International Register of Systematic Reviews (ID: CRD42024572063) [8]. A systematic search of PubMed, Web of Science, and Google Scholar was performed in August 2024. Screening and selection of studies were carried out using Rayyan [9]. The following keywords were applied: *direct oral anticoagulants, vitamin K antagonists, cognitive function, atrial fibrillation, DOACs, VKAs, cognitive impairment, cognitive decline, quality of life*, and *anticoagulation therapy*.

### Study selection

We included all studies meeting the following criteria: (1) population: patients diagnosed with AF; (2) intervention: DOACs (e.g., dabigatran, rivaroxaban, apixaban); (3) comparator: VKAs (e.g., warfarin); (4) outcomes: changes in cognitive function, incidence of cognitive impairment, progression of cognitive decline, quality of life; and (5) study design: randomized controlled trials (RCTs), cohort studies, and observational studies. We excluded the following: (1) patients without atrial fibrillation or those with other primary conditions affecting cognitive function, patients previously diagnosed with dementia, valvular AF; (2) anticoagulants other than DOACs or studies not directly comparing DOACs and VKAs; (3) studies lacking a comparative group of VKAs; (4) studies not reporting cognitive function outcomes or related quality of life measures; and (5) the study was of the following types: case reports, case series, review articles, and non-comparative studies.

### Data extraction

Three reviewers independently assessed the titles, abstracts, and full-text papers of the identified articles for inclusion, and any duplicates were removed using the Rayyan web tool [9]. All searches followed a two-phase screening strategy. Phase one assessed the eligibility of the title and abstract of every manuscript generated from the electronic searches against the predetermined inclusion and exclusion criteria. Studies that either did not meet the inclusion criteria or met at least one exclusion criterion were excluded at this phase. In Phase 2, the full-text papers were retrieved for the articles identified in Stage 1 and assessed against the predetermined inclusion and exclusion criteria. Data extraction was performed for the following variables: study design, sample size, patient demographics (age, sex), comorbidities, type of intervention, comparison group, duration of treatment, cognitive function assessment methods, baseline cognitive function, follow-up duration, changes in mental function scores, incidence of cognitive impairment or dementia, quality of life measures, adverse events related to anticoagulation therapy, and CHA_2_DS_2_-VASc (congestive heart failure, hypertension, age ≥75 years, diabetes mellitus, prior stroke or transient ischemic attack, vascular disease, age 65–74 years, sex category) score.

### Quality assessment

The Cochrane Risk of Bias tool for randomized controlled trials (RoB 2) and the Methodological Index for Non-Randomized Studies (MINORS) were used to assess study quality for both prospective and retrospective cohort studies [10,11]. For cross-sectional studies, the AXIS tool was applied. Two reviewers independently performed the quality assessment for each article, with disagreements resolved through discussion or, if necessary, by involving a third reviewer. Following this process, 11 studies published between 2018 and 2024 were included in the systematic review and meta-analysis [12–22].

### Statistical analysis

Descriptive analyses of study and participant characteristics were conducted using Microsoft Excel. Categorical variables (e.g., study design, intervention type, outcome measures) were summarized as frequencies and percentages, while continuous variables were summarized as means with standard deviations.

Meta-analyses were performed using Review Manager (RevMan) version 5.4, applying a random-effects model to account for inter-study variability. Effect estimates for dichotomous outcomes were expressed as risk ratios (RRs) with 95% confidence intervals (CIs). Heterogeneity was evaluated using the I^2^ statistic and interpreted as low (0%–25%), moderate (26%–50%), high (51%–75%), or substantial (>75%). All statistical tests were two-sided, and *P* values < .05 were considered statistically significant.

## RESULTS

### Study selection and screening

**Figure 1:**
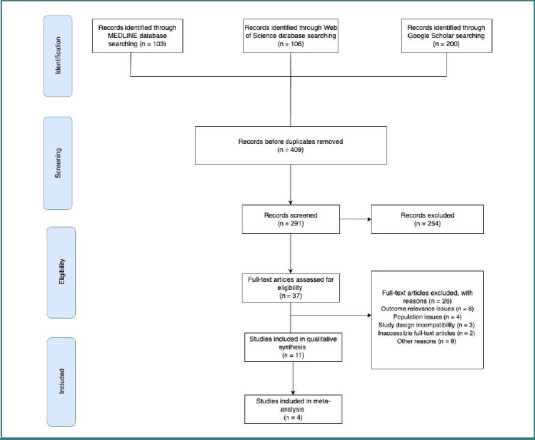
PRISMA flowchart depicting the study selection process

A comprehensive search of PubMed, Web of Science, and Google Scholar yielded 409 records during the identification phase (103 from PubMed, 106 from Web of Science, and 200 from Google Scholar). After removing duplicates, 291 records remained for title and abstract screening (Screening Phase), of which 254 were excluded for irrelevance or ineligibility. In the eligibility phase, 37 full-text articles were reviewed, and 26 were excluded for the following reasons: outcome relevance issues (*n* = 8), population issues (*n* = 4), study design incompatibility (*n* = 3), inaccessible texts (*n* = 2), and other reasons (*n* = 9), such as the use of inverse probability weighting (IPW/IPTW) or the absence of direct comparisons between DOACs and VKAs. The PRISMA flow diagram illustrates the study selection process (Figure 1).

### Study characteristics

Cohort studies were the most common study design, comprising 63.6% (*n* = 7) of the included studies. These consisted of retrospective cohort studies (*n* = 3), a population-based cohort study (*n* = 1), a historical cohort study (*n* = 1), and a propensity score–matched cohort study (*n* = 1). One multicentre, prospective, observational, longitudinal cohort study (Strawberry study) was included. Randomized controlled trials accounted for 18.2% (*n* = 2) and included the GIRAF randomized clinical trial and a prospective, open-label vanguard clinical study with blinded endpoints. The remaining studies consisted of an observational, cross-sectional, multicenter study (9.1%) and a prospective, parallel group, randomized, open, masked outcome assessment study (9.1%). The included studies originated from diverse geographic regions: United States contributed with the most significant number (*n* = 3, 27.3%), followed by Taiwan (*n* = 1), United Kingdom (*n* = 1), Brazil (*n* = 1), Hobart, Tasmania, Australia (*n* = 1), Japan (*n* = 1), and Madrid, Spain (*n* = 1). Table 1 summarizes the basic demographics and characteristics of the included studies. The primary outcomes focused on the incidence of all-cause dementia, including subtypes such as vascular dementia, Alzheimer's disease, and unspecified dementia. Secondary outcomes included stroke incidence, transient ischemic attack (TIA), intracerebral bleeding, myocardial infarction (MI), and mortality. Several studies evaluated cognitive performance using tools like the Montreal Cognitive Assessment (MoCA) and Clinical Dementia Rating (CDR). Other studies assessed time to stroke, cardiovascular events, or death. Additional outcomes included composite measures of bleeding (BARC 2-5), stroke, and death, as well as analyses of frailty, dependency, and cognitive impairment in elderly atrial fibrillation patients.

**Table 1:** Basic study characteristics and participants’ demographics

Study ID	Name of Journal Published in	Study Design	Country	Sample size (*n*)		Mean age		Men		Women	
				DOAC	VKA	DOAC	VKA	DOAC	VKA	DOAC	VKA
Sagris, 2023 [1]	European Journal of Internal Medicine	Retrospective observational study	United States	117,960	146,111	70.3 ± 11.9	70 ± 12.3	45,026 (41.8%)	45,190 (41.7%)	NR	NR
Hsu, 2021 [2]	Journal of the American Heart Association	Population-based cohort study	Taiwan	17,065	8,024	70.3 ± 11.7	70.4 ± 11.6	3,592	3,560	2,442	2,474
Cadogan, 2021 [3]	British Medical Journal (BMJ)	Historical cohort study	United Kingdom	18,513	20,687	NR	NR	10,267	11,444	8,246	9,243
Caramelli, 2022 [4]	BMC medicine	GIRAF randomized clinical trial	Brazil	83	66	74	76	51	39	32	27
Chen, 2018 [5]	Journal of the American Heart Association	Propensity score–matched cohorts	United States	31,304	39,202	67–69	68–73	35–37%	39–40%	35–45%	35–45%
Bunch, 2022 [6]	Journal of Arrhythmia	Randomized, prospective, open-label study	Salt Lake City, USA	50	51	73.4 ± 5.5	74.0 ± 6.5	26 (52.0%)	28 (54.9%)	NR	NR
Bezabhe, 2022 [7]	Journal of the American Heart Association	Retrospective cohort study	Hobart, Australia	4,191	NR	73.0 ± 10.4	NR	NR	NR	NR	NR
Thunell, 2024 [8]	American Academy of Neurology	Retrospective cohort	USA	0	0	NR	NR	NR	NR	NR	NR
Mostaza, 2018 [9]	Journal of Geriatric Cardiology	Observational, cross-sectional study	Madrid, Spain	0	0	83 ± 5.0	NR	NR	NR	NR	NR
Kirchhof, 2018 [10]	European Heart Journal	Prospective, parallel-group study	Europe & North America	318	315	NR	NR	100 (31%)	109 (35%)	NR	NR
Saji, 2018 [11]	BMJ	Multicentre, prospective cohort study	Japan	240	160	NR	NR	NR	NR	NR	NR

DOAC, Direct Oral Anticoagulants; VKA, Vitamin K Antagonists; NR, Not Reported. Mean age values are presented as mean ± standard deviation (SD).

### Patient characteristics

The included studies analyzed a total of 1,839,604 patients, divided into Group 1 (DOAC users) and Group 2 (warfarin users). For the 5-year follow-up, the sample size was 215,404 (DOACs: 117,960; warfarin: 146,111), while for the 10-year follow-up, 19,208 patients were included (DOACs: 9,604; warfarin: 81,082). The overall mean age of participants was 72.1 years (SD ± 11.6), ranging from 40 to 84 years. The mean age in the DOAC group was 71.8 years (SD ± 11.5) compared to 72.4 years (SD ± 11.7) in the warfarin group. Men accounted for approximately 52% of all participants, while women comprised 48%, with slight variations between the two groups. In the DOAC group, men comprised 41-52% and women 35-45%. The warfarin group showed a similar distribution, with men at 41-53% and women at 34-45%. The most common comorbidities were hypertension (86-91%), diabetes mellitus (12-44%), and heart failure (20-62%), with heart failure and chronic kidney disease (7-32%) slightly more prevalent in the warfarin group. Dyslipidemia affected 57-73% of patients, while vascular disease and cerebral infarction were reported in 13-29% and 7-19%, respectively. Depression and chronic obstructive pulmonary disease (COPD) were prevalent in 25-32% and 6-28% of participants, respectively.

### Intervention details

The studies compared DOACs (dabigatran, apixaban, rivaroxaban, and edoxaban) with VKAs (warfarin) across varying treatment durations. Follow-up periods ranged from 3 to 6 months for short-term interventions to longer durations of 24 months, with extended follow-up spanning 5 to 10 years. One study reported a mean treatment duration of 332 days for DOACs versus 316 days for warfarin, whereas another required a minimum treatment period of at least 3 months for inclusion.

### Outcome measures

#### Cognitive function assessment methods

Cognitive function was assessed using standardized tools, with the MoCA and Mini-Mental State Examination (MMSE) being the most frequently used, reported in 54.5% (*n* = 6/11) of studies. The Clinical Dementia Rating (CDR) was applied in 18.18% (*n* = 2/11). At the same time, detailed neuropsychological batteries, including the Trail Making Test, Boston Naming Test (BNT), and Digit Symbol Substitution Test (DSST), were also used in 18.18% (*n* = 2/11) of the cases.

Pfeiffer's Test for Cognitive Impairment and computer-generated neuropsychological tests (CGNT), assessing reaction time and attention, were each reported in 9% (*n* = 1/11) of the studies. Additional tools, such as the Alzheimer's Disease Assessment Scale (ADAS) and Hachinski Ischemic Score (HIS), were each used in 9% (*n* = 1/11) of studies. Table 2 provides an overview of the intervention details and outcomes measured.

**Table 2:** Intervention details and outcome measures in studies comparing DOACs and VKAs in patients with atrial fibrillation

Study ID	Intervention	Comparison	Duration	Cognitive Function Methods	Baseline Cognitive Function	Cognitive Function Changes	Incidence of Dementia	Quality of life	Adverse events	CHA_2_DS_2_-VASc Score
Sagris, 2023 [1]	DOAC	VKA	5 years, 10 years	NR	NR	NR	DOAC: 3.3% (10 years), VKA: 4.7%; HR 0.72 (95% CI: 0.62–0.83)	NR	Intracerebral Bleeding: DOAC (0.9%) vs VKA (1.4%); Death: DOAC (17.6%) vs VKA (25.1%)	NR
Hsu, 2021 [2]	DOAC	VKA	90 days	NR	NR	NR	Dementia: DOAC (304) vs VKA (360); Lower risk observed in DOAC with high bleeding risk	NR	NR	2.9 (DOAC), 3.0 (VKA)
Cadogan, 2021 [3]	DOAC	VKA	NR	NR	NR	NR	GP-recorded Dementia: 3.2%; MCI Diagnosis: DOAC users lower	NR	NR	NR
Caramelli, 2022 [4]	DOAC (Dabigatran)	VKA	24 months	MoCA, MMSE, NTB, CGNT	NR	Dabigatran: MoCA decline (-0.96); significant difference favoring VKA	No patients developed dementia	NR	Deaths: 14 (Dabigatran: 5; Warfarin: 9); Stroke: 1 in Warfarin group	4 (both groups)
Chen, 2018 [5]	DOAC (Dabigatran, Rivaroxaban, Apixaban)	VKA	NR	NR	NR	NR	Dementia cases: Warfarin-Dabigatran: 739 vs 724; Warfarin-Rivaroxaban: 944 vs 648; Warfarin-Apixaban: 474 vs 277	NR	NR	3.0–4.3
Bunch, 2022 [6]	DOAC	VKA	24 months	MMSE, HIS, ADAS	Dabigatran: MMSE 27.4 ± 2.7; Warfarin: MMSE 27.7 ± 2.2	Identical cognitive scoring; No patients developed dementia	Dementia: 0; Cognitive Impairment: Dabigatran 15.6%, Warfarin 6.5%	MLHFQ, Anti-Clot Treatment Scale	NR	≥2
Bezabhe, 2022 [7]	OAC	Non-OAC, Warfarin	Matched cohort	NR	NR	NR	OAC Dementia Rate: 4.4/1000 person-years (95% CI: 3.4–5.6); Non-OAC: 7.5/1000 person-years (95% CI: 6.2–9.0)	NR	NR	2.9 ± 1.4
Thunell, 2024 [8]	DOAC (Dabigatran, Rivaroxaban, Apixaban, Edoxaban)	VKA	332 days (DOAC), 316 (VKA)	NR	NR	NR	Dementia incidence within 3 years: Declined from 13% (2007) to 11.39% (2017)	NR	NR	NR
Mostaza, 2018 [9]	DOAC	VKA	≥3 months	Pfeiffer’s Test	NR	Median cognitive decline: 1.9 ± 2.1	32.3% of patients showed cognitive impairment	NR	NR	5.0 ± 1.4
Kirchhof, 2018 [10]	DOAC	VKA	3 months, 30 days	MoCA	Median MoCA: 27.0 (25.0–29.0)	Change: DOAC 0.0 (-1.0, 2.0); VKA 1.0 (-1.0, 2.0); MoCA decline greater in VKA	Mild cognitive dysfunction decreased by 7.2%	EQ-5D, SF-12, Karnofsky Status	Major Bleeding: DOAC (6.2%) vs VKA (7.9%); Stroke/TIA: DOAC (0.6%), VKA (0%)	2.4 (1.2)
Saji, 2018 [11]	DOAC	VKA	36 months	CDR, MMSE, MoCA	MMSE (20–30), CDR (0–0.5)	NR	NR	ADL scale, Barthel Index, Lawton and Brody	NR	NR

DOAC, Direct Oral Anticoagulants; VKA, Vitamin K Antagonists, OAC, Oral Anticoagulants; NR, Not Reported. Cognitive function methods include MoCA (Montreal Cognitive Assessment), MMSE (Mini-Mental State Examination), and NTB (Neuropsychological Test Battery).

#### Baseline cognitive function

Baseline cognitive function, assessed using the MMSE, was similar between groups, with scores of 27.4 ± 2.7 for DOAC users and 27.7 ± 2.2 for warfarin users. Median MMSE scores were 27.0 (IQR 25.0-29.0) across all patients. Additional scores included ADAS (13.7 ± 5.8 for DOACs and 13.1 ± 5.7 for warfarin) and HIS (1.36–1.44), indicating comparable baseline cognitive performance between the groups.

#### Changes in cognitive function scores

Cognitive function changes were minimal and comparable between groups. The MMSE, NTB, and CGNT scores showed no statistically significant differences, though the MoCA score favored the warfarin group (mean difference: -0.96). At the end of the study, the median MoCA score remained 28.0 (IQR = 26.0–29.0) in both groups. For DOAC users, abnormal MoCA scores (<26) decreased by 5.1%, whereas the reduction was 9.2% in the warfarin group. Cognitive decline was limited, with slight differences in specific scores favoring warfarin.

#### Incidence of cognitive impairment or dementia

At 5 years, all-cause dementia was diagnosed in 3.9% of patients in both DOAC and VKA groups (HR = 1.01; 95% CI, 0.96–1.05). By 10 years, the incidence of dementia was lower in the DOAC group (3.3%) compared to the VKA group (4.7%; HR = 0.72; 95% CI, 0.62–0.83). Specific comparisons showed fewer dementia cases with DOACs, including 724 vs. 739 for dabigatran, 648 vs. 944 for rivaroxaban, and 277 vs. 474 for apixaban. Cognitive dysfunction at baseline improved by 7.2% at follow-up, suggesting a slight benefit with DOACs over time.

#### Quality of life measures

A limited number of studies assessed quality of life. Tools used included the Minnesota Living with Heart Failure Scale, the Anti-Clot Treatment Scale Quality of Life Survey, and the EQ-5D, SF-12, and Karnofsky Performance Status. These measures evaluated overall health status, treatment satisfaction, and functional performance, though reporting across studies was inconsistent.

#### Adverse events related to anticoagulation therapy

DOAC treatment was associated with a lower risk of adverse events compared to VKAs, particularly in long-term follow-up. At five years, DOACs significantly reduced the risk of intracerebral bleeding (0.9% vs. 1.4%, DOAC: 997/106,000, VKA: 1,515/106,000) and death (17.6% vs. 25.1%, DOAC: 19,003, VKA: 27,171), with a marginal reduction in ischemic stroke (8.0% vs. 8.3%). At 10 years, DOACs continued to show benefits, reducing the risk of vascular dementia (0.7% vs. 1.2%, DOAC: 70/9600, VKA: 113/9600) and death (23.2% vs. 34.2%, DOAC: 2250, VKA: 3307). Additional events included significant bleeding (6.2% in DOACs vs. 7.9% in VKAs), stroke/TIA (0.6% in DOACs vs. 0% in VKAs), and a small number of cardiovascular and non-cardiovascular deaths.

#### CHA_2_DS_2_-VASc score

The mean CHA_2_DS_2_-VASc score was comparable between DOAC and VKA groups, ranging from 2.9 to 4.3 across studies. In the MarketScan dataset, scores for DOAC users ranged from 3.1 to 3.4, while those for VKA users ranged from 3.0 to 3.4. In the Optum dataset, slightly higher scores were reported, with DOAC and VKA users ranging between 3.6 and 4.3. Matched cohorts showed a mean score of 2.9 ± 1.4 for OAC users. One study reported a higher average score of 5.0 ± 1.4, indicating an elevated stroke risk. Both groups demonstrated moderate to high bleeding risk with minimal differences in CHA_2_DS_2_-VASc scores.

### Meta-analysis

**Figure 2:**
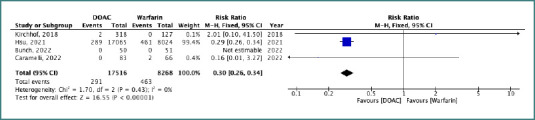
Forest plot comparing the risk of stroke or systemic embolism (SSE) between DOACs and VKAs

**Figure 3:**
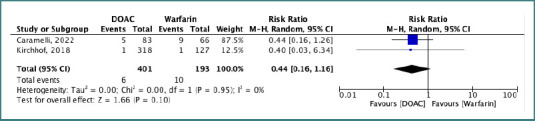
Forest plot comparing the risk of all-cause death between DOACs and VKAs

The pooled risk ratio (RR) for stroke or systemic embolism (SSE) was 0.30 (95% CI, 0.26–0.34), indicating a significantly lower risk with DOACs compared with VKAs. Heterogeneity was negligible (I^2^ = 0%, *P* = 0.43), and the result was statistically significant (*P* < 0.00001) (Figure 2). For all-cause mortality, the pooled RR was 0.44 (95% CI, 0.16–1.16), indicating no statistically significant difference between DOACs and VKAs (*P* = 0.10). Heterogeneity was low (I^2^ = 0%, *P* = 0.95) (Figure 3).

## DISCUSSION

This systematic review and meta-analysis compared the effects of DOACs and VKAs on the cognitive function of patients with AF. Our findings suggest that patients treated with DOACs demonstrate better cognitive outcomes than those receiving warfarin, particularly over long-term follow-up. This benefit may be attributable to the more stable anticoagulant effect of DOACs, which reduces the risk of silent strokes and intracranial hemorrhage, thereby supporting cognitive preservation [16]. In addition, the favorable pharmacokinetic profile of DOACs may further contribute to these benefits. Unlike VKAs, which inhibit vitamin K-dependent pathways and promote vascular calcification, DOACs selectively target specific coagulation factors without adversely affecting vascular health, thus lowering the risk of chronic ischemic changes [12].

Additionally, a lower risk of dementia in AF patients and reduced risk of vascular dementia were noted in previously published sources that reported fewer vascular complications and intracranial bleeding in DOAC users. These outcomes are critical in preserving cognitive function, as vascular damage significantly contributes to cognitive decline [18-24]. This significant reduction highlights the potential neuroprotective benefits of DOACs, which may be attributed to their ability to provide consistent anticoagulation, thereby reducing the risk of microembolic events and cerebral microbleeds [24]. These findings highlight the advantages of DOACs in minimizing cerebrovascular damage and suggest that DOACs may offer potential benefits for preserving cognitive function in AF patients, particularly over longer periods.

Our findings yield results similar to those reported in previous articles. A systematic review and meta-analysis by the American College of Cardiology Foundation showed lower dementia risk with using DOACs over warfarin in AF patients. Edoxaban had the highest rank, but there was no statistically significant data. The study also focused on Asian populations, with patients' ages ranging from 65 to 75 years old [25]. Compared to our analysis, a more diverse population primarily addresses all causes of dementia, with secondary outcomes including stroke, TIA, MI, intracerebral bleeding, and mortality across more geographic regions. Moreover, a systematic review with meta-analysis reported that DOAC therapy in AF patients resulted in a significant reduction in dementia risk over VKA. The findings were predominantly observed in men. However, it focused specifically on dementia risk, with age groups ranging from 65.9 to 86.1 and a median follow-up period spanning 243 days to 9 years [26].

In contrast, our findings indicated a broader age range, from 40 to 84 years, and a follow-up period ranging from 3 to 6 months to 10 years, allowing for a wider objective evaluation of cognitive outcomes beyond dementia. By encompassing a wider age range and longer follow-up duration, our research captures a more diverse population. It may offer additional insights into the longitudinal effects of anticoagulation therapy on cognitive health. Additionally, a cohort study done by Grymonprez *et al*. reported a significant reduction in dementia risk and cognitive decline, particularly with the use of apixaban and edoxaban, compared to VKAs in AF patients. However, the study primarily focused on new-onset dementia and its subtypes. It faced challenges such as potential misclassification due to observational design, the incursion of death as a competing risk, and a relatively short mean follow-up duration of 1.3 years, extending up to 6 years [27].

In comparison, our findings include a more extended follow-up period of up to 10 years, providing a more comprehensive evaluation of cognitive outcomes over time. Another meta-analysis, conducted in the Department of Cardiovascular Medicine, a second affiliated hospital of Nanchang University, yielded similar results. This analysis used CHA_2_DS_2_-VASc scores as a key factor in assessing the benefits of anticoagulants, with higher scores associated with reduced dementia risk when using DOACs compared to VKAs. The studies focused on variability in efficacy and analyzed each DOAC subgroup, concentrating primarily on the incidence of dementia [2]. In comparison, our analysis shows both dementia risk and broader cognitive outcomes. A systematic review and meta-analysis conducted at the Cardiovascular Institute, Dongzhimen Hospital, Beijing University of Chinese Medicine, similarly evaluated the effects of oral anticoagulants on clinical outcomes in AF patients. Both analyzed outcomes of ischemic stroke, major bleeding, and all-cause mortality. The study targeted AF patients with dementia or cognitive impairment with a shorter follow-up duration of 1-4 years [3]. In contrast, our analysis revealed the outcomes of a general AF patient with a longer follow-up period, extending from 3 to 6 months to 10 years.

There are several limitations in our study. First, the strength and reliability of our results depend heavily on the quality of the included studies. Variations in study design, sample sizes, follow-up durations, and the measurement of cognitive function could have led to variations in our overall results. We did our best to minimize bias by using thorough search strategies and established evaluation tools, such as RoB2 and MINORS; however, publication bias remains a risk. Studies with null or negative results may have been less represented, which could distort the outcomes. Additionally, our analysis was limited to studies published in English. This means we might have missed valuable insights from research in other languages that could have expanded our understanding of the connection between anticoagulant use and cognitive function.

Lastly, our combined data may oversimplify the complex relationships between genetic predispositions, treatment adherence, lifestyle choices, and other factors that affect cognitive outcomes in AF patients. Despite these limitations, our study has significant strengths. We present a comprehensive overview of the mental health effects of VKAs and DOACs by integrating data from several high-quality trials conducted in various locations. A balanced analysis is made possible by combining observational studies and RCTs, which combine controlled experimental outcomes with practical, real-world relevance. The rigorous adherence to PRISMA guidelines and thorough quality assessment methods strengthens the reliability of our findings. This study highlights crucial areas for further research to improve the understanding of cognitive outcomes in AF patients. Future studies should focus on assessing the long-term effects and seeking long-term insights into anticoagulant therapy and mental health using patient-reported outcomes.

Moreover, conducting meta-analyses using individual patient data (IPD) could provide valuable insights into how age, comorbidities, and genetic variations influence cognitive outcomes. To build a more comprehensive evidence base, international collaborations are needed to address language barriers and publication bias. It is also crucial to clarify the biological mechanisms underlying the apparent neuroprotective effects of DOACs. Such knowledge would support the development of more tailored treatment strategies.

Given the rapid growth of research in this area and the increasing use of DOACs, it is essential to continuously update the literature on their potential neuroprotective benefits. Incorporating the latest evidence into clinical guidelines will help ensure that anticoagulant therapy decisions consider not only stroke prevention but also long-term cognitive health.

## CONCLUSION

We conducted a systematic review and meta-analysis to compare the impact of DOACs and VKAs on cognitive function in patients with AF. Overall, cognitive decline was minimal and similar between groups; however, DOAC users demonstrated a lower incidence of all-cause dementia and fewer adverse events, particularly during long-term follow-up. These findings suggest that DOACs may offer a safer clinical profile and a modest cognitive advantage over VKAs.

Future studies should further evaluate the long-term effects of anticoagulant therapy on cognitive outcomes, incorporating patient-reported measures to capture real-world impacts on mental health and quality of life. In addition, meta-analyses using individual patient data are needed to clarify how factors such as age, comorbidities, and genetic variations influence cognitive trajectories in anticoagulated patients.
